# Idiopathic chronic cough: a real disease or a failure of diagnosis?

**DOI:** 10.1186/1745-9974-1-9

**Published:** 2005-09-23

**Authors:** LPA McGarvey

**Affiliations:** 1 Department of Medicine, The Queen's University of Belfast, Grosvenor Road, Belfast BT126BJ, N Ireland, UK

**Keywords:** Cough, idiopathic, diagnostic protocol

## Abstract

Despite extensive diagnostic evaluation and numerous treatment trials, a number of patients remain troubled by a chronic and uncontrollable cough. Eosinophilic bronchitis, atopic cough and non-acid reflux have been recently added to the diagnostic spectrum for chronic cough. In some cases, failure to consider these conditions may explain treatment failure. However, a subset of patients with persisting symptoms may be regarded as having an idiopathic cough. These individuals are most commonly female, of postmenopausal age and frequently report viral upper respiratory tract infections as an initiating event. This paper seeks to explore the validity of idiopathic cough as a distinct clinical entity.

## Introduction

Despite considerable advance in the understanding of cough, the effective management of patients with a chronic cough can be difficult. For the patient, a cough which persists can be associated with considerable distress and impaired quality of life [1]. For the physician, failure to obtain a treatment response may lead to the mistaken belief that the cough is functional or psychogenic. There are a number of reasons why the cough may be difficult to treat. In some cases it may reflect an inadequate approach to diagnostic evaluation and failure to appreciate both pulmonary and extra pulmonary causes for chronic cough [2,3]. In other cases, trials of therapy may be of inadequate dose and of insufficient duration. However, an alternative explanation is that a distinct diagnostic entity exists, namely idiopathic cough [4]. If this is the case then almost nothing is known about the underlying pathophysiological processes responsible for this condition and at present there are no effective treatment options. This article seeks to examine the evidence for idiopathic cough as either a distinct diagnosis or simply the result of incomplete evaluation and inadequate courses of therapy.

## Diagnostic protocols for chronic cough

The term 'idiopathic' comes from the Greek word *idiopatheia *and is defined in the Oxford English Dictionary as a 'disease not preceded or occasioned by another, or by any known cause' [5]. In the original description of cough evaluation and management by Irwin and colleagues, idiopathic cough was not described and indeed treatment failure was extremely rare [6]. Using a stepwise approach known as the anatomic diagnostic protocol, Irwin and colleagues reported that a cause for cough could be determined successfully in up to 98% of cases and was due to either cough variant asthma (CVA), rhinosinusitis associated with postnasal drip syndrome (PNDS) or gastro-oesophageal reflux disease (GORD) [6]. The subsequent experience from this group [7,8] and a number of others in hospital-based settings [9,10] has remained the same and the diagnostic protocol has been recommended by the American College of Chest Physicians in their clinical guidelines for the management of cough [11].

Although the systematic evaluation of both extrapulmonary and pulmonary causes for cough is widely held to be effective, doubt has been cast on the perception that the diagnostic triad of CVA, PNDS and GORD accounts for the almost all causes of chronic cough [12,13]. Despite adopting a comprehensive evaluation of patients referred with cough, many groups have reported diagnostic and treatment failure in anything from 12 – 42% of patients [14-16]. For some, this represents a population with idiopathic cough [16] but others suggest it reflects failed management [17]. Specifically, the failure to prescribe sedating antihistamines for postnasal drip syndromes [17] and the inadequate treatment of gastro-oesophageal reflux disease have been highlighted [18].

There are a number of possible explanations for the impressive treatment response described by Irwin and others. Firstly, it is probable that the original referral populations included patients with cough following a viral upper respiratory infection. It is now recognised that cough following an upper respiratory tract infection may persist beyond three weeks and only resolve spontaneously some weeks or months later. Therefore some of the 'treatment success' may merely have reflected the natural resolution of a prolonged post-viral cough. Secondly, many patients were prescribed older generation antihistamines, which have an imprecise pharmacological action but presumably exert most of their antitussive effect by crossing the blood-brain barrier and acting directly on the cough control centre within the brain. Crucially, response to such therapy tells us little about the aetiology of the cough. Finally, these original studies reported on short-term treatment outcomes and provided little information on the long-term treatment response. Initial treatment benefit may well diminish over time and the timing of patient follow-up may explain some of the variance in outcome described by different centres [19].

## Failure to adequately treat cough

Current guidelines have recommended a combination of diagnostic testing and empirical trials in the management chronic cough [20]. Some authors have reported that the characteristics of a cough confer little diagnostic information [21] but in practice, prominent symptoms of an upper airway disorder or indigestion should prompt a treatment trial of anti-rhinitic therapy or anti-reflux therapy [20]. The question of how much and for how long of a specific treatment has yet to be unequivocally answered. This point is perhaps best illustrated in the management of GORD associated cough. Although lacking a strong evidence base, it may be necessary to embark on intensive courses of anti-reflux therapy, because in contrast to the symptoms of heartburn, which usually resolve after a few days treatment, improvement in cough seems to take much longer [18,22]. In one study, mean duration to treatment success was 179 days [18]. As a consequence, failure to comply with prolonged therapy and lifestyle changes may result in relapse and explain poor treatment success even in patients with a high suspicion of GORD associated cough [19].

Alternatively, some individuals on relatively high doses of acid suppression may exhibit relative proton pump therapy resistance. This is particularly the case with attempts to suppress proximal and laryngophayngeal reflux where despite single and higher dose treatment regimes, 44% of patients demonstrated abnormal levels of acid exposure on simultaneous oesophagel and laryngeal pH testing [23]. Finally, a minority of patients who fail adequate courses of acid suppressive therapy may ultimately require anti-reflux surgery [24]. This final observation has contributed to the growing appreciation that acid may not be the sole aggravating factor in gastric refluxate. Until recently, this concept of 'non-acid reflux' as a cause for cough had been infrequently considered. It will be discussed together with a number of other 'new causes for cough' in the subsequent section of this review.

## New causes for cough

Given the extent of associated literature, it is barely conceivable that any respiratory physician is unaware of the most common associations with chronic cough, namely asthma, GORD and rhinosinusitis, more recently termed upper airway cough syndrome. In the last decade, a series of important observations have led to the appreciation of new diagnostic possibilities. Most importantly, the application of induced sputum in the evaluation of cough has led to the identification of eosinophilic airway syndromes [25]. These conditions are characterized by the presence of eosinophilic airway inflammation but crucially the absence of the airway dysfunction (airflow variability or bronchial hyperreactivity) normally attributed to asthma. The best-described condition is eosinophilic bronchitis, which may account for up to 15% of patients referred to hospital with chronic cough [26]. It frequently responds to inhaled corticosteroids, and as these are often prescribed empirically in the community the exact prevalence of this condition is unknown. More recently, a number of Japanese groups have described a syndrome of "Atopic Cough" [27]. These patients are atopic, have an isolated bronchodilator resistant cough and an eosinophilic tracheobronchitis. Like eosinophilic bronchitis, there is no evidence of airway hyperreactivity but in contrast, the cough does not respond to inhaled corticosteroids. Without adequate attention to the inflammatory characteristics of the airway, and reluctance to prescribe inhaled steroids to patients with normal airway function then either of these syndromes may be incorrectly labeled as having an idiopathic cough.

The concept of 'Non-acid reflux' has recently gained attention. Irwin and colleagues [24] reported on a group of 8 patients that had persistent cough despite total or near total acid suppression utilizing proton pump inhibitors, prokinetic agents and antireflux diet (omeprazole 20–80 mg p.o. daily and cisapride 40–80 mg p.o. daily). These 8 patients had 24 hour ambulatory oesophageal pH monitoring while on medical therapy, and in all patients the % of 24 hours spent at pH < 4.0 was zero or near zero. Despite this, all 8 patients underwent antireflux surgery with marked reduction in cough scores after surgery, which were maintained after 12 months of follow up. This study suggests antireflux surgery may improve cough that is resistant to medical therapy, and that the improvement is sustained. Acid reflux disease in patients with cough and GORD may be a misnomer since non-acid reflux may be responsible for cough in some patients (volume reflux with gastric enzymes, bile salts etc.) [28]. Thus failure to respond to antireflux therapy may not indicate an idiopathic chronic cough.

Finally an association between cough, GORD and a familial sensory neuropathy has recently been reported [29]. The locus for the particular gene appears to be located on chromosone 3. In a series of personal communications with other cough specialists, it would appear similar associations have been encountered suggesting such clinical features may represent a new cough syndrome.

The common and less common associations with cough must be rigorously excluded before a diagnosis of idiopathic cough can be assigned. None-the-less, this author firmly believes such a condition exists and it will be addressed in some detail in the following section.

## Idiopathic cough as a distinct clinical entity

The accumulation of experience and information regarding idiopathic cough suggests a fairly well defined population of patients. The over-representation of women in the specialist cough clinic referral population is widely acknowledged, and the preponderance of females among idiopathic coughers is particularly striking. Some centers have reported female prevalence rates of more than 80% [14-16,30-33] (See table 1). Gender differences in health-related quality of life and as a consequence differences in health seeking behaviour is one explanation [34] but others have suggested a distinct clinical phenotype [4]. Typically the female patients are of peri- or post menopausal age, report a preceding upper respiratory tract infection (URTI) and have a heightened cough reflex to tussive stimuli [16]. These observations raise the possibility that sex hormones and viral URTIs in some way contribute to the development of an idiopathic cough in susceptible individuals.

**Table 1:** Characteristics of idiopathic cough patients attending specialist cough clinics

	Number (% female)	Mean age (SD) (years)	Median cough duration (range) (months)
O'Connell F *et al *[14]	16(81%)	51(31–70)*	72 (12–240)
McGarvey L *et al *[15]	8(75%)	46(8)	19 (6–36)
Forsythe P *et al *[30]	6(66%)	47(13)	72(2–240)
Jatakanon A *et al *[31]	10(50%)	60(4)	60 (18)^
Birring SS et al [32]	25(72%)	55(3)	12 (7–360)
Chaudhuri R *et al *[33]	6(60%)	58(9)	14(19)^
Haque R *et al *[16]	31(76%)	57(32–81)*	72 (8–324)*

*Data given as median (range), ^Data given as mean (SD)

## Possible mechanisms for idiopathic cough

The human cough reflex consists of an afferent arm comprising cough receptors, afferent pathways, central processing and an efferent pathway. The cough reflex can be modified at any point along this reflex and unraveling the mechanisms responsible is key to a more complete understanding of cough pathophysiology and its successful treatment. Afferent sensory nerves are not static entities and the term 'plasticity' has been used to describe changes in function contributing to the sensitization that occurs in response to various stimuli, in particular those associated with airway inflammatory processes [35]. Although viral infections are a major cause of cough and appear to be frequently reported in patients with idiopathic cough, little is known regarding the effects of viruses on cough sensitivity. Following respiratory syncytial virus infection in rats, tachykinin content within the lung is increased [36] along with an upregulation in the substance P receptor, neurokinin (NK) 1 [37]. These changes appear to persist for some time after the virus is cleared. In guinea pigs, inoculation with the Sendai virus has been associated with a qualitative change in airway sensory nerves whereby nonnociceptive neurons express tachykinins [38]. This 'phenotypic switch' is one plausible mechanism whereby viral infection causes increased tachykinergic content in airway nerves which possibly contribute to persistent reflex hypersensitivity and cough. It is unknown if such processes occur in man, but abnormal intraepithelial nerves containing increased neuropeptide content have been reported in bronchial biopsies from patients with idiopathic cough [39].

Only a few studies have specifically commented on findings in the airways of patients with idiopathic cough. Birring et al. observed a mild chronic lymphocytic airway inflammation in a predominately female population of idiopathic coughers and highlighted the striking association with organ specific autoimmune disease in particular hypothyroidism [40]. They suggested that the presence of increased lymphocytes within the airway reflected either an aberrant homing of lymphocytes from the primary site of autoimmune inflammation to the lung or a distinct autoimmune process within the lungs [40]. A more recent study has confirmed the dominance of lymphocytes in the airways of females with idiopathic cough. In this study, significantly elevated numbers of activated CD4+ lymphocytes were noted in bronchoalveolar lavage fluid from menopausal women with isolated dry cough compared to matched controls. This group hypothesized that menopausal effects on lymphocyte activation within the airway may lead to disordered responses to airway insults such as infection [41].

Gender and sex hormones may have important effects on neuro-immune events within the airway. A number of studies have demonstrated a heightened cough reflex sensitivity in females compared to males both in healthy individuals [42,43] and cough subjects [44]. This gender difference has not been observed in children, raising the possibility that sex hormones may influence the reflex [45]. Women of post-menopausal age appear to have a heightened cough reflex although this has not been consistently demonstrated [46]. None-the-less, oestrogen levels begin to decrease around the time of the menopause, which may exert an effect on cough reflex sensitivity. Danazol, a synthetic androgen that decreases oestrogen levels, has been shown to inhibit the upregulation of the cough reflex observed in female guinea pigs following treatment with an ACE-inhibitor [47].

## Conclusion

Although inadequate management will continue to explain a significant number of patients with a chronic and uncontrollable cough, an attempt has been made in this article to highlight idiopathic cough as a distinct clinical entity. Although without firm evidence, idiopathic cough may arise as a consequence of the persisting effects of viral infection or other noxious aggravants in susceptible individuals. The excess of middle-aged females with idiopathic cough raises the possibility of some sex hormonal influence. Precision in this area will be greatly hampered unless further research is undertaken.
